# Should the Minimal Intervention Principle Be Considered When Investigating Dual-Tasking Effects on Postural Control?

**DOI:** 10.3390/brainsci10010001

**Published:** 2019-12-19

**Authors:** Felix Wachholz, Federico Tiribello, Arunee Promsri, Peter Federolf

**Affiliations:** 1 Department of Sport Science, University of Innsbruck, Innsbruck 6020, Austria; federico.tiribello@student.uibk.ac.at (F.T.); arunee.promsri@student.uibk.ac.at (A.P.); peter.federolf@uibk.ac.at (P.F.); 2 Department of Physical Therapy, University of Phayao, Phayao 56000, Thailand

**Keywords:** motor control, automatization, optimal feedback control, minimal intervention principle, principal component analysis, postural control, adolescents and adults, attentional focus

## Abstract

Dual-tasking charges the sensorimotor system with performing two tasks simultaneously. Center of pressure (COP) analysis reveals the postural control that is altered during dual-tasking, but may not reveal the underlying neural mechanisms. In the current study, we hypothesized that the minimal intervention principle (MIP) provides a concept by which dual-tasking effects on the organization and prioritization of postural control can be predicted. Postural movements of 23 adolescents (age 12.7 ± 1.3; 8 females) and 15 adults (26.9 ± 2.3) were measured in a bipedal stance with eyes open, eyes closed and eyes open while performing a dual-task using a force plate and 39 reflective markers. COP data was analyzed by calculating the mean velocity, standard deviation and amplitude of displacement. Kinematic data was examined by performing a principal component analysis (PCA) and extracting postural movement components. Two variables were determined to investigate changes in amplitude (*aVar_k_*) and in control (*N_k_*) of the principal movement components. Results in *aVar_k_* and in *N_k_* agreed well with the predicted dual-tasking effects. Thus, the current study corroborates the notion that the MIP should be considered when investigating postural control under dual-tasking conditions.

## 1. Introduction

Human motor control is largely automatized in many every-day situations, which means that sensorimotor processing requires little or no attention [1]. The so-called dual-task paradigm has been used by researchers to investigate this connection between postural control and attentional focus [2,3]. Interestingly, results are not always consistent when evaluating the effect of performing a concurrent cognitive task on the neural control of stabilizing one’s posture. Some researchers argue that postural control is controlled in a more effective way during dual-tasking [4,5,6,7,8], suggesting that automatic processes regulate postural control [1,3,4,9]. However, others have found traditional variables like center of pressure (COP) sway variability, velocity, frequency, or area to be increased and interpret this as less effective postural control [10,11,12,13,14,15] resulting from cognitive resource competition [10]. With regard to different sensory situations, closed eyes in a bipedal stance usually produce an increase in the sway [16,17,18], arguably due to missing sensory information provided by vision. There is much debate about the differences in postural control between adolescents and adults. Some researchers report that postural control is well developed around the age of 12 [19,20] whereas others report differences in equilibrium scores until the age of 15–16 [21,22].

An often-cited model for motor control is the optimal feedback control (OFC) theory, which predicts the minimal intervention principle (MIP) [23]. The MIP suggests that the controller focuses more on the task-relevant dimensions in the control of different movement dimensions, while more variability is tolerated in movement dimensions that are not task-relevant [23,24,25]. “Movement dimensions” in this context relate to the degrees of freedom in the human body, enabled by joints between the segments [26,27] and to the coordinative patterns that emerge in the control of these degrees of freedom. A suitable approach to evaluate human postural control is to analyze kinematic data using principal component analysis (PCA), which reveals different movement strategies [28,29,30,31]. In an unconstrained bipedal stance with eyes open, the most dominant movement component is the anterior-posterior ankle sway (ankle strategy) with an approximately 70% overall share of the postural variance [29,31,32,33]. Research investigating postural control by measuring muscle activity using electromyography [34] or joint torques [35] support the ankle strategy as the dominant coordinative pattern in the bipedal stance.

Trying to stand as still as possible is an unusual task, since performing several tasks simultaneously while standing occurs often in daily life situations [10,36]. Focusing on standing as still as possible directs a large proportion of attention to the motor control task. In this sense, a single-task “standing as quiet as possible” exercise leads to much less automatized postural control [3,10]. We hypothesized that focusing one’s attention on postural control with an explicit instruction to “move as little as possible” can override neural mechanisms such as the MIP. If, however, attention is drawn away from the task of balancing, e.g., towards a concurrent cognitive task, then we expect that the control of postural movements will be more automatized, and that the MIP will play a more important role in the automatized control.

The main purpose of the current study was to better understand the effect of dual-tasking on postural control. We hypothesized that the MIP needs to be considered when assessing the effects of dual-tasking on postural control, which would imply that divergent effects should be expected between different movements’ dimensions (coordinative patterns within the postural movements). More specifically, we hypothesized that a more automatized neural control would prioritize specific movement components that threaten postural stability, such as the anterior-posterior ankle strategy in a normal bipedal stance. Such an upregulation in control may lead to reduced movement amplitude and increased frequency of control interventions (changes in acceleration). In contrast, the more automatized neural controller might de-prioritize higher order movement components, which are less critical for maintaining stability. This de-prioritization may lead to an increase in the movement amplitude and a decrease in the frequency of control interventions. In movement dimensions that are largely irrelevant for maintaining stability, such as lateral sway in bipedal standing (where stability is provided mainly by the two legs), we did not expect to observe an effect of dual-tasking, since stability in these movement components does not fully rely on a permanent neuromuscular control. We distinguished between adult participants and adolescents, since automatization of postural control might not yet be fully developed in the latter. Finally, as a comparison, we also evaluated COP movements.

## 2. Materials and Methods

### 2.1. Ethics

All participants were informed about the measurement procedures, about any possible risks involved and provided written informed consent regarding their participation. Participants were free to withdraw from the experiment at any time without reason. Prior to any measurements, the study had been approved by the Board for Ethical Questions in Science of the University of Innsbruck (Certificate 09/2018). All measurements and the use of equipment and procedures were performed in accordance with the Declaration of Helsinki (1964).

### 2.2. Participants

A total of 38 volunteers (23 adolescents, 15 adults) were included in the current study (Table 1). Participants were recruited from local sport clubs and represented a healthy and above-average physically active population. Diagnosed injuries, such as concussion or neurological disorders within the last six months, as well as known problems in joints, tendons or muscles were set as exclusion criteria.

### 2.3. Measurement Procedures

The participants were instructed to stand as steady as possible on a marked area on the ground, in a hip-wide stance, with their hands on the hips. Three different balance trials were analyzed. The first trial, standing in a bipedal stance with eyes open, lasted 60 s and participants were asked to focus their gaze on a cross placed 5.5m in front of them at a height of 1.75 m. The second trial, standing in a bipedal stance with eyes closed, lasted 30 s. The third trial lasted 60 s and was again a hip-wide, bipedal, eyes-open trial, in which participants were asked to count backwards in steps of two starting from a random three-digits number (e.g., 374, 372, 370, …). Counting backwards in steps of two was chosen for the dual-task condition since it is a continuous task that is difficult enough to permanently require cognitive resources, provoking an automatized postural control, but it is not so difficult that it might disturb postural control [10]. Participants were asked to count as fast and correctly as possible. The number of answers and of mistakes were recorded to encourage participants to try their best, however, for the purpose of the current study the actual cognitive performance was not further analyzed. Between trials, participants had breaks of at least 90 s in which they could move freely around the room. The order of the trials was not randomized since, on the one hand, we did not anticipate fatigue effects, and, on the other hand, we could not rule out that participants might silently repeat a similar cognitive task (silently counting) if a trial without instructions followed a dual-tasking trial. By performing the single-task balancing trials first, we hoped to avoid preconditioning the participants.

### 2.4. Instrumentation

The motion of the COP was recorded at 3 kHz using a ground-embedded AMTI force plate (AMTI, Watertown, USA). Synchronized with the force plate, kinematic data quantifying postural movements of the body were collected at 250 Hz using an 8-camera Vicon motion tracking system (Vicon Motion Systems Ltd., Oxford, UK) and 39 retro-reflective markers attached to the volunteers at anatomical landmarks on the skin or on tight sport-clothing using double sided tape. Markers on the head and wrists were attached using modified sweatbands. The marker model was based on the full-body Plug-In Gait marker model provided by Vicon. Force plate and kinematic data were synchronously collected using the Vicon software (Vicon Nexus, Version 2.2.3; Vicon Motion Systems Ltd., Oxford, UK).

### 2.5. Data Processing

MatLab R2019b (The Mathworks Inc. Natick, MA, USA) was used to process the data from the force plate, as well as the kinematic motion tracking data. In all trials, the first five 5 s were omitted to avoid “settling-in” effects and the following 25 s were selected for the analysis.

### 2.6. Processing of COP Data

The center of pressure (COP) data was down sampled to 250 Hz to have equal frequencies for further analysis. The force plate recorded COP data, which was processed in the anterior-posterior and medio-lateral direction. The standard deviation of COP displacement, the COP mean velocity and the amplitude of COP displacement were calculated [37]. To normalize and enable comparisons between participants of different size, COP data was normalized using the statokinesigram method [38]. The used code for calculating the variables using MatLab can be found in the Supplementary Material.

### 2.7. Processing of Kinematic Data: Calculation of Principal Movements (PMs)

The kinematic data was analyzed using a principal component analysis (PCA) to evaluate postural movements, i.e., changes in the postural configuration [29]. All of the following PCA-based calculations were computed using a software package called “PManalyzer” [39], which is provided through open access. As a first analysis step, gaps in the marker trajectories were filled [40,41], then the data from each trial was normalized by subtracting the mean posture and dividing by the trial’s mean Euclidean distance [29,32], finally, the marker coordinates were weighed according to the relative body mass, which they represent [29,42]. This normalization was designed to remove anthropometric differences while preserving the relative amplitude of the marker movements and to ensure that each participant equally affected the PCA output [29,32]. Hence, after the normalization, the data from all trials and all subjects could be concatenated into one single input matrix for the PCA [28,30,43,44]. Therefore, the PCA resulted in one set of eigenvalues *EV_k_* and one set of eigenvectors *PC_k_* (*k* represents the order index), which were common to all participants and all trials. These *PC_k_* form an orthonormal basis in which postural movements can be quantitatively compared between subjects. Then, the normalized data from each trial were projected onto the *PC_k_*-basis vectors, which yield subject- and trial-specific scores *PP_k_(t*), where *PP* stands for principal (postural) position and each value of the *PP*-time series indicates how much the posture at time *t* deviates from the mean posture according to the movement pattern defined by the associated *PC_k_* vector [28,32,45].

Together, the *PC_k_* vector and the *PP_k_* time series can be called a principal movement (*PM_k_*), which is an analogy for other variables that are often used to describe human movement, for instance, the “knee movement” is also defined through an algorithm that encodes how the “knee angle” is calculated from the available information (in the *PM_k_* the *PC_k_* vectors provide this algorithm) and the actual evolution of the variable during the trial (knee angle(*t*); *PP_k_(t*)). This analogy can be further expanded: by (double) differentiation, one obtains the angular velocity (angular acceleration) of the knee angle, and similarly, one can calculate the principal velocity *PV_k_* and principal acceleration *PA_k_* of each *PM_k_* [29], thus quantifying the rate and acceleration at which postural changes occur. The *PM_k_* thus provide a set of variables (whole-body movement patterns) that quantify the specific task/movement for which they were calculated. From a (bio-)mechanical point of view, the *PM_k_* were validated by demonstrating that an independently measured kinematic variable, the center of pressure movement COP(t), can be predicted from the *PM_k_* with very high precision [29].

### 2.8. Dependent PM-Based Variables

In addition to the eigenvalues *EV_k_*, which were introduced above and which allow comparison to many other studies [28,30,31,43,44,45], we also calculated the absolute variance (*aVar_k_*) of each *PP_k_(t*), which quantifies the subject- and trial-specific variance represented by each postural movement component. The variable *aVar_k_* provides a measure for the amplitude of the associated movement component observed during each balancing trial.

In order to investigate alterations in neuromuscular control, we determined the number of zero crossings (*N_k_*) in the *PA_k_* time series [45]. Each postural acceleration *PA_k_* is the result of the interplay between agonistic and antagonistic muscle groups. A large number of zero crossings suggests a tightly controlled movement that is frequently adjusted by the sensorimotor system. A decline in *N_k_* could be an indication that the processing time for adjustments increases, or it could be an indication that a movement component is not controlled as tightly, e.g., because the system attributes less priority to the movement component. A number of previous studies have already used this variable and found that it is sensitive, for example, to age differences or to leg dominance [45,46,47].

### 2.9. Statistics

Shapiro Wilk Tests were used to test for normal distribution in the dependent variables. Three different trials were measured for each participant. Therefore, to asses for overall differences between the trials, a repeated, measured analysis of variance (rANOVA) was applied. Group (adolescents/adult) was considered as a between-subject factor. Since a separate rANOVA was calculated for each of the first 10 PMs, the Holm-Bonferroni correction [48] was applied to correct for a familywise accumulation of alpha errors. Then, *p*-values were ranked from lowest to highest and compared to a Holm-Bonferroni adjusted alpha level, which were calculated using the following equation:

$$\mathrm{Holm}\;\mathrm{Bonferroni}\;=\;\frac{\mathrm{target}\;\alpha \mathrm{lpha}\;\mathrm{level}\;(0.05)}{n-\mathrm{rank}\;\mathrm{number}\;\mathrm{of}\;\mathrm{pair}\;(\mathrm{by}\;\mathrm{degree}\;\mathrm{of}\;\mathrm{significance})+1}$$

If the Holm-Bonferroni corrected rANOVA indicated a significant difference, then a post-hoc test was performed to determine which trial(s) differed from the other(s). Here a normal Bonferroni-correction was applied.

If data were not normally distributed, then Friedmann tests with Dunn-Bonferroni post-hoc tests were performed. All statistical testing was conducted using SPSS (IBM SPSS Statistics, Version 24, SPSS Inc., Chicago, IL, USA).

## 3. Results

### 3.1. Center of Pressure Motion

**Figure 1:**
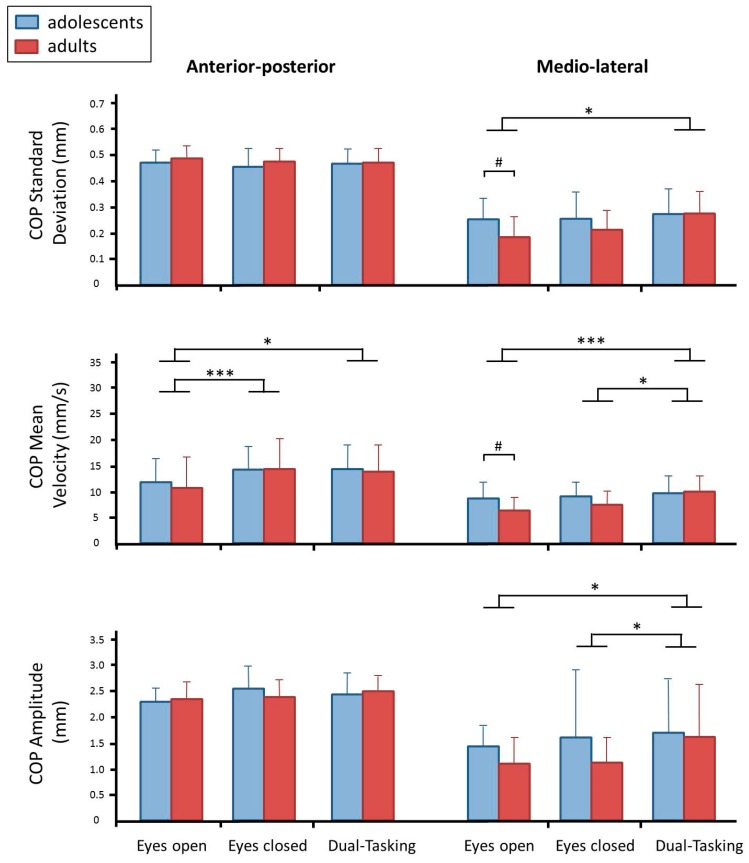
Characterization of the center of pressure (COP) motion: normalized standard deviation (SD), mean velocity, and amplitude. The anterior-posterior motion is shown on the left, the medio-lateral on the right. Blue bars represent the adolescents, red bars represent the adults (mean ± standard error). Asterisks indicate significance between trials (*p* < 0.001 ***, *p* < 0.05 *) and hashtags between groups (*p* < 0.05 #).

In the anterior-posterior direction (Figure 1, column 1), differences were only observed in the mean velocity of the COP motion (*F*(2, 72) = 11.107, *p* < 0.001, *η*^2^ = 0.236). Post-hoc tests showed that the COP mean velocity was smaller in the eyes-open trial compared to the eyes-closed trial (*p* < 0.001) and to the dual-tasking trials (*p* = 0.003).

Also, in the medio-lateral direction (Figure 1, column 2), differences were found in the mean velocity (*F*(2, 72) = 13.796, *p* < 0.001, *η*^2^ = 0.277). The mean velocities of COP were faster during dual-tasking compared to the eyes-open situation (*p* < 0.001) and compared to standing with eyes-closed (*p* < 0.008). Moreover, the standard deviation of the COP (quantifying medio-lateral sway) differed between trials *F*(2, 72) = 5.809, *p* = 0.005, *η*^2^ = 0.139. In the eyes-open trial, the standard deviation was smaller than during dual-tasking (*p* = 0.004). The amplitude of COP presented similar results, showing larger amplitude in dual-task trials than in eyes-open (*p* = 0.048) and eyes-closed trials (*p* = 0.048).

Group effects appeared in two variables: the adolescents had larger standard deviations and faster mean velocities in the medio-lateral COP motion in the eyes-open trials (*t*(36) = 2.592, *p* = 0.014 and *t*(36) = 2.714, *p* = 0.010, respectively).

### 3.2. Amplitude of Postural Movement Components

**Figure 2:**
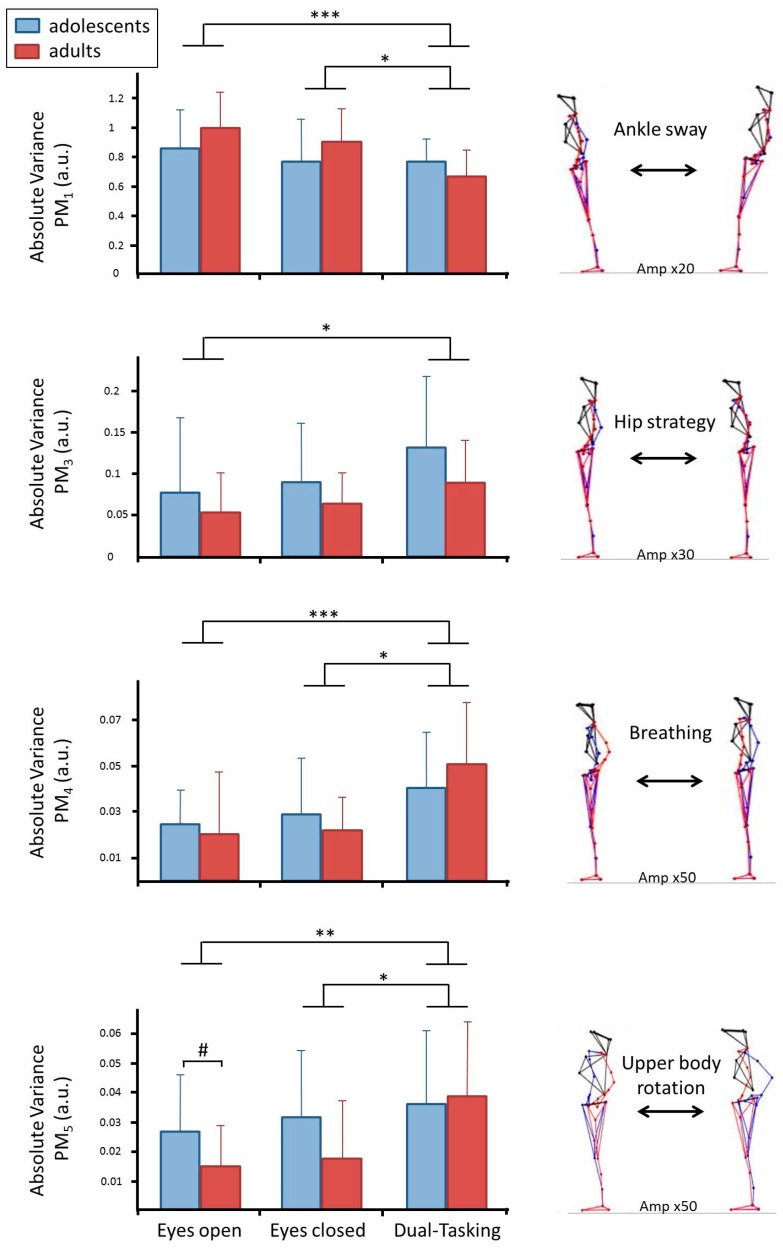
Results for *PM_1_*, *PM_3_*, *PM_4_*, *PM_5_* in variable *aVar_k_*. Lines with asterisks (*p* < 0.001 ***; *p* = 0.001 **; *p* < 0.05 *) indicate significant differences between trials and hashtags between groups (*p* < 0.05 #). Blue bars represent adolescents, red bars adults (mean ± standard error). A visualization of the movement component through their extreme positions is included on the right. For better clarity, the deviation from the mean posture is amplified by the specified factor *Amp*.

The first ten principal components explained 97.67% of the total postural variance. Video files provided in the Supplementary Materials visualize the movement components. Table 2 presents the eigenvalues *EV_k_* of each component and offers a qualitative description of the main aspects that each *PM_k_* represented. The table also includes the *p*-values obtained from rANOVAS (*F*) or Friedmann tests (*X*^2^) calculated on the absolute variances *aVar_k_* for each *PM_k_*, and lists the rank and Holm-Bonferroni adjusted alpha-level. Table 3 lists the results of the post-hoc analysis. In summary, we observed that dual-tasking caused a decrease in movement amplitude in *PM_1_*, but an increase (or no changes) in the higher order movement components (Figure 2).

### 3.3. Control of Postural Movement Components

**Figure 3:**
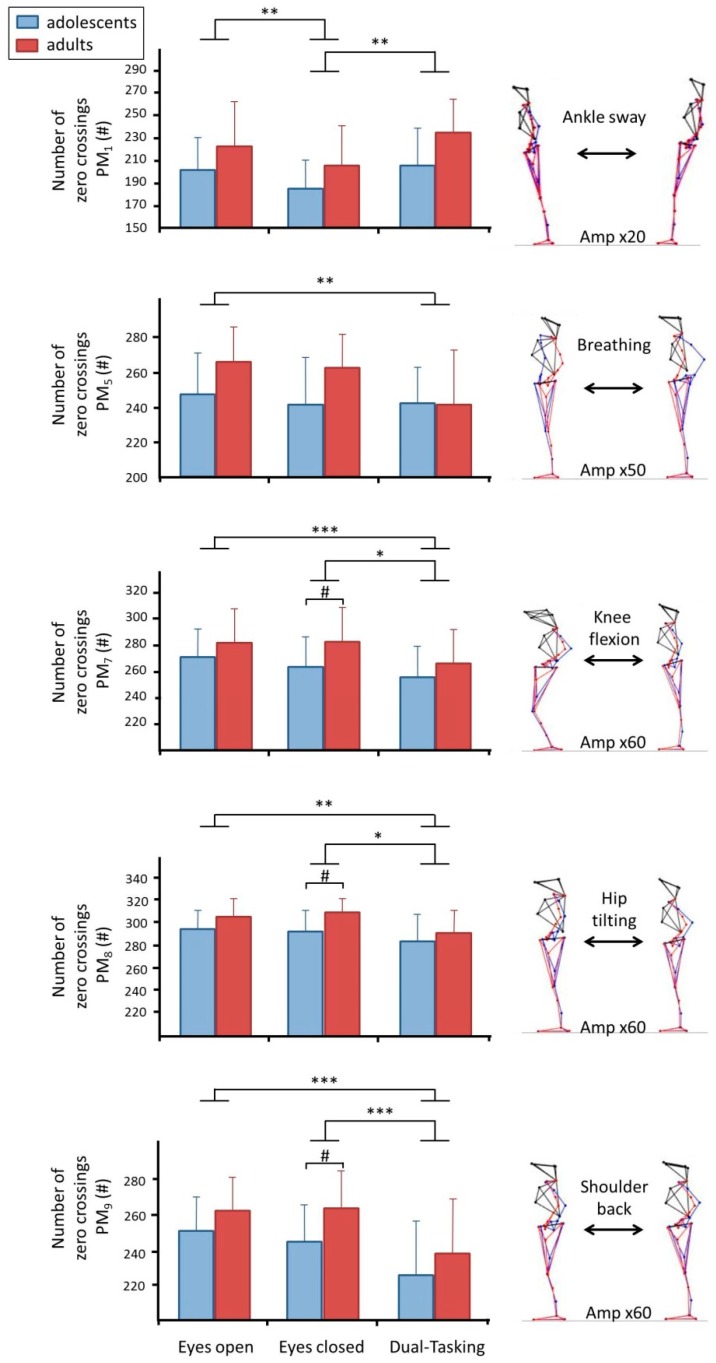
Results for *PM_1_*, *PM_5_*, *PM_7_*, *PM_8_*, *PM_9_* in variable *N_k_*. Lines with asterisks (*p* < 0.001 ***; *p* = 0.001 **; *p* < 0.05 *) indicate significant differences between trials and hashtags between groups (*p* < 0.05 #). Blue bars represent adolescents, red bars represent adults (mean ± standard error). A visualization of the movement component through their extreme positions is included on the right. For better clarity, the deviation from the mean posture is amplified by the specified factor *Amp*.

Within the first 10 *PM_k_*, significant trial effects in *N_k_* were found in *PM_1_, PM_5_, PM_7_, PM_8_* and *PM_9_*. (Table 4 and Figure 3). In *PM_1_*, *N_1_* was smaller in the eyes-closed trials compared to eyes-open (*p* = 0.001) and dual-tasking trials (*p* = 0.001). In all higher order components, we observed either no differences (*PM_2_, PM_3_, PM_4_, PM_6_, PM_10_,*) or that *N_k_* decreased in the dual-tasking trial compared to the eyes-open trial (*PM_5_)* or compared to both the eyes-open and the eyes-closed trials (*PM_7_, PM_8_*, *PM_9_*). Group differences were (after Holm-Bonferroni correction) found in *PM_7_* (*t*(36) = 2.568, *p* = 0.015), *PM_8_* (*t*(36) = 3.114, *p* = 0.004) and *PM_9_* (*t*(36) = 2.871, *p* = 0.007) with adults showing greater *N_k_* than adolescents, however, these differences occurred only in the eyes-closed trial.

## 4. Discussion

### 4.1. Main Results

The current study investigated dual-tasking effects, and hypothesized that the MIP needs to be considered. We argued that this implies divergent effects in different movement dimensions/components, depending on the relevance of the movement component for the task of maintaining stability. Specifically, we predicted for dual-tasking that anterior-posterior ankle sway, which is claimed to be of high importance for postural control [29,31,32] and was represented in *PM_1_*, would decrease in amplitude (*aVar_k_*) while the frequency of control interventions (*N_k_*) would increase [45,46,47]. Our results supported the first prediction (*aVar_1_* decreased), but did not confirm the second prediction (*N_1_* was not significantly different from eyes-open standing and the difference to *N_1_* in the eyes-closed trial is most likely an effect of the system coping with reduced sensory information in the eyes-closed trial [17]). For higher-order, anterior-posterior movement components, e.g., *PM_3_*, *PM_7_*, *PM_8_*, *PM_9_*, and *PM_10_*, we had predicted a de-prioritization in the dual-tasking condition, which would manifest as an increase in movement amplitude and a decrease in *N_k_*. Our results corroborated the amplitude prediction in *PM_3_*, *PM_8_*, *PM_9_*, and *PM_10_* and the *N_k_* prediction in *PM_7_*, *PM_8_*, and *PM_9_*. Finally, we expected that lateral ankle sway, which was represented in *PM_2_*, would not be affected by dual-tasking. Our results also corroborated this assumption, as no significant differences were found in *aVar_2_* or *N_2_*. In summary, the results of the current study supported many of the various predictions. In contrast, no result was observed that directly contradicts the hypothesis of the minimum intervention principle [23] influencing how neural control of posture changes as attention is drawn away from the balancing task to a concurrent cognitive task.

In the current study we also hypothesized that adults would show clearer effects of dual-tasking since automatized postural control might be more refined compared to the still developing adolescents [19,20,21,22]. At first glance, several of the graphs in Figure 2 and Figure 3 seem to support this supposition—the dual-tasking effect seems to be driven more by the adult group than by the adolescent group, which is in line with previous research [9,49]. However, overall the statistical results could not support this assumption—most likely due to insufficient power. Differences between adults and adolescents in neural control of postural movements, and how this control may be affected by dual-tasking need to be investigated in future studies.

A curious result was observed in the COP data: our hypothesis predicted effects in the anterior-posterior movement components whereas we expected no effects on the medio-lateral sway—predictions that were largely corroborated when assessing the actual kinematic movement patterns. The COP motion, however, showed no effect on anterior-posterior movement amplitude, only on the anterior-posterior component of the COP velocity. In contrast, significant increases in medio-lateral COP movement amplitude and COP velocity were found for dual-tasking. The results for the anterior-posterior direction might be explained by a cancelation effect: the movement amplitude of *PM_1_* decreased, but the movement amplitude of higher order movement components increased. It appears that these opposing effects canceled out their effect on the anterior-posterior COP motion. The increase in medio-lateral COP amplitude might be explained by the fact that the more complex higher-order movement components are less pure-plane motions than the lower-order ankle sway movement components. The increases that were observed in the amplitude of higher-order movement components may therefore affect medio-lateral COP motion. However, in the medio-lateral direction there is no opposing effect of a lower-order ankle sway that would diminish the effect on COP motion. Hence, an increase in medio-lateral COP motion amplitude was detectable.

According to the literature, COP presents larger sway in dual-tasking in both directions, the anterior-posterior [4,5] and medio-lateral axis [6]. These results and the observations of the COP motion presented here thus support the notion that postural movement patterns should be investigated in postural control research. Analyzing COP motion may not be sufficient to fully understand the changes that take place in the neural control of postural stability.

The current pilot study used the MIP as a theoretical framework to explain the effects of dual-tasking on postural control, and to our knowledge, presented the first application where the MIP was used to predict differences between single- and dual-tasking. Focusing on postural control in a bipedal stance confirmed the existing results concerning human movement strategies, but also helped to gather new insights into how movement control appears to be organized by the postural control system. Future studies should consider the MIP as an underlying mechanism and help to deepen the understanding of motor control hierarchies.

### 4.2. Limitations

The relatively small sample size is a limitation of the current study, especially after dividing into two groups. On the one hand, we could observe highly significant differences in *aVar_k_* variance and *N_k_* in the principal components, however, with small effect sizes (Table 3 and Table 4), resulting in small statistical power. On the other hand, the trend in differences seen between adolescents and adults might be significant if the study was repeated with a larger sample.

A third limitation is that the analysis was restricted to the first ten PMs. This restriction implies an approximation of the “real” postural movements, which can only be fully reconstructed from the whole set of PMs.

The non-randomized order of the trials might be seen as limitation; however, we did not expect the participants to fatigue, as we provided them at least 90 s of rest after every trial. Additionally, we hoped to prevent a preconditioning of the participants as mentioned in the Methods section.

## 5. Conclusions

The current study hypothesized that the MIP provides a concept by which dual-tasking effects on the organization and prioritization of postural control can be predicted. The experimental results were in good agreement with these predictions. Therefore, we suggest that the MIP should be considered in future investigations of postural control under dual-tasking conditions.

## Figures and Tables

**Table 1:** Anthropometric information about the participants; mean ± SD; BMI = Body Mass Index.

Participants	Age (years)	Body Height (cm)	Body Weight (kg)	BMI
All (*n* = 38)	18.3 ± 7.2	171.7 ± 14.3	63.8 ± 16.5	21.1 ± 2.8
Adolescents (*n* = 23)	12.7 ± 1.3	163.6 ± 12.1	54.0 ± 13.1	19.8 ± 2.8
Female (*n* = 8)	11.5 ± 1.3	152.7 ± 9.4	41.9 ± 9.9	17.7 ± 2.4
Male (*n* = 15)	13.3 ± 0.8	169.5 ± 9.0	60.3 ± 10.3	20.9 ± 2.4
Adults Male (*n* = 15)	26.9 ± 2.3	183.9 ± 7.3	78.9 ± 7.3	23.3 ± 1.1

**Table 2:** Eigenvalues *EV_k_*, a qualitative description of each principal movement (PM), the applied statistics (*X*^2^ for Friedmann and *F* for rANOVA), the calculated *p*-value to determine significant differences in *aVar_k_* between trials, the resulting rank, and the adjusted alpha-level resulting from the Holm-Bonferroni correction are displayed. Significant *p*-values are printed in bold.

PM_k_	EV_k_ (%)	Qualitative Description of PM_k_	Statistics	*p*-Value	Rank	Holm-Correction of Alpha-Level
**PM_1_**	63.75	Ankle sway anterior-posterior	*F*	**0.000010**	2	0.0056
**PM_2_**	14.77	Hip sway medio-lateral	*X* ^2^	0.035362	9	0.0250
**PM_3_**	7.29	Hip flexion anterior-posterior	*X* ^2^	**0.002479**	7	0.0125
**PM_4_**	2.67	Frontal plane trunk rotation	*X* ^2^	**0.000120**	3	0.0063
**PM_5_**	2.40	Breathing	*X* ^2^	**0.000683**	6	0.0100
**PM_6_**	2.09	Breathing and head movement	*X* ^2^	0.030197	8	0.0167
**PM_7_**	1.07	Knee flexion anterior-posterior	*X* ^2^	0.056789	10	0.0500
**PM_8_**	0.77	Hip tilting anterior-posterior	*X* ^2^	**0.000294**	5	0.0083
**PM_9_**	0.69	Bringing shoulders back	*X* ^2^	**0.000156**	4	0.0071
**PM_10_**	0.58	Shifting legs anterior-posterior	*X* ^2^	**0.000004**	1	0.0050

**Table 3:** Statistical results for the differences between trials in the *aVar_k_* of *PM_k_* based on rANOVA (*F*) and Friedmann test (*X*^2^). The degrees of freedom (DoF) and *p*-values are reported, as well as the post-hoc results with *p*-values and Rosenthal’s *r* as effect size (*r*). Asterisks indicate significant difference (*p* < 0.001 ***; *p* = 0.001 **; *p* < 0.05 *). Abbreviations: EO—eyes-open; EC—eyes-closed; DT—dual-task.

	Overall Trial Effect on Variance			Post-Hoc			
PM_k_	*F*|*X*^2^	DoF	*p*-Value	EO–DT	*r*	EC–DT	*r*
**PM_1_**	13.623	2, 72	0.001 ***	0.001 ***	-	0.016 *	-
**PM_3_**	12.0	2	0.002 *	0.002 *	0.128	0.117	0.077
**PM_4_**	18.053	2	0.001 ***	0.001 ***	0.149	0.004 *	0.120
**PM_5_**	14.579	2	0.001 *	0.001 **	0.132	0.009 *	0.111
**PM_8_**	16.262	2	0.001 ***	0.001 ***	0.141	0.002 *	0.115
**PM_9_**	17.526	2	0.001 ***	0.001 ***	0.141	0.002 *	0.128
**PM_10_**	24.789	2	0.001 ***	0.001 ***	0.166	0.001 ***	0.153

**Table 4:** Results of the statistical analysis evaluating trial differences in the variable *N_k_* within the first 10 PMs. All data were normally distributed and thus the results are based on rANOVAs. The table lists the *F*-values, degrees of freedom (DoF), *p*-values, partial eta-squared *η*^2^, and where applicable the significance of pairwise post-hoc comparisons. Asterisks indicate significant results after applying the Holm-Bonferroni correction (*p* < 0.001 ***; *p* = 0.001 **; *p* < 0.05 *). Post-hoc tests revealed differences between eyes-open (EO), eyes-closed (EC) and dual-tasking (DT) trials.

	Overall Trial Effect on NoZC				Post-Hoc		
PM_k_	*F*	DoF	*p*-Value	*η* ^2^	EO–EC	EO–DT	EC–DT
**PM_1_**	10.813	1.707, 61.437	0.001 ***	0.231	0.001 **	0.490	0.001 *
**PM_2_**	1.079	2, 72	0.345	0.029			
**PM_3_**	0.501	2, 72	0.608	0.014			
**PM_4_**	4.267	2, 72	0.018 *	0.106			
**PM_5_**	8.360	1.695, 61.016	0.001 *	0.188	0.342	0.001 **	0.083
**PM_6_**	0.797	1.633, 58.779	0.433	0.022			
**PM_7_**	9.444	2, 72	0.001 ***	0.208	1.000	0.001 ***	0.010 *
**PM_8_**	11.176	1.711, 61,607	0.001 ***	0.237	1.000	0.005 *	0.001 *
**PM_9_**	19.233	1.349, 48.580	0.001 ***	0.348	1.000	0.001 ***	0.001 ***
**PM_10_**	2.248	2, 72	0.113	0.059			

## Supplementary Materials

The following are available online at https://www.mdpi.com/2076-3425/10/1/1/s1, Video S1: A video visualizing the analyzed PMs 1–5, Video S2 visualizes PMs 6–10, Code S3 is the code used for calculation of the COP variables.
